# Risk factors for recurrent positive results of the nucleic acid amplification test for COVID-19 patients: a retrospective study

**DOI:** 10.1007/s13577-021-00618-9

**Published:** 2021-09-23

**Authors:** Wanwan Yi, Xuan Long, Jin Liu, LiShuai Shi, Zichen Chen, Jing Yang, Ziyu Yang, Zhongwei Lv, Hengwei Fan

**Affiliations:** 1 Department of Nuclear Medicine, Shanghai Tenth People’s Hospital, Tongji University School of Medicine, Yanchang Middle Road 301, Shanghai, 20072 China; 2 Department of Respiratory Diseases, Shanghai Tenth People’s Hospital, Tongji University School of Medicine, Yanchang Middle Road 301, Shanghai, 20072 China; 3 Department of Hepatic Surgery, The Eastern Hepatobiliary Surgery Hospital, Navy Medical University (Second Military Medical University), Yangpu Road 225, Shanghai, 200438 China; 4 Department of Integrated Chinese and Western Medicine, The Eastern Hepatobiliary Surgery Hospital, Navy Medical University (Second Military Medical University), Shanghai, 200438 China; 5 Department of Infection Medicine, Guanggu District, Maternal and Child Health Hospital of Hubei Province, Wuhan, 430070 Hubei China; 6 Department of Standardized Training Base for Resident Physicians, Shanghai Tenth People’s Hospital of Tongji University, Shanghai, China; 7 Department of Chemistry and Pharmacy, Guangxi Normal University, Guangxi, 541004 China

**Keywords:** COVID-19, SARS-CoV-2, Nucleic acid amplification tests, Positive retest, Negative retest

## Abstract

Positive retests of COVID-19 represent a public health concern because of the increased risk of transmission. This study explored whether factors other than the nucleic acid amplification test (NAAT) contribute to positive retest results. Patients with COVID-19 admitted to the Guanggu district of the Hubei Maternal and Child Health Hospital between February 17 and March 28, 2020, were retrospectively included. The patients were grouped into the negative (*n* = 133) and positive (*n* = 51) retest groups. The results showed that the proportion of patients presenting with cough was higher (*P* < 0.001) and the proportion of patients with dyspnea was lower (*P* = 0.018) in the positive than in the negative retest group. The positive retest group showed shorter durations between symptom onset and hospitalization (*P* < 0.001) and symptom onset and the first positive NAAT (*P* = 0.033). The positive retest group had higher basophil counts (*P* = 0.023) and direct bilirubin (*P* = 0.032) and chlorine concentrations (*P* = 0.023) but lower potassium concentrations (*P* = 0.001) than the negative retest group. Multivariable regression analysis showed that coughing (OR = 7.59, 95% CI 2.28–25.32, *P* = 0.001) and serum chloride concentrations (OR = 1.38, 95% CI 1.08–1.77, *P* = 0.010) were independently associated with a positive retest result. Coughing and serum chloride concentrations were independent risk factors for positive NAAT retest results. Patients with a hospital stay of < 2 weeks or a short incubation period should stay in isolation and be monitored to reduce transmission. These results could help identify patients who require closer surveillance.

## Introduction

Coronavirus disease 2019 (COVID-19), caused by SARS-CoV-2, is extremely contagious, and it spreads rapidly globally; it had affected > 210 million people and caused > 4.5 million deaths as of September, 2021 [1]. The common signs of COVID-19 include fever, cough, and shortness of breath [2]. Several cases are mild, but the mortality rate is high for patients who develop the critical form of the disease [3, 4]. The infection has no recognized treatment [2–4], but vaccines may help control it [5–7].

The key to optimal COVID-19 management is the identification of positive cases. Such cases should be isolated to prevent the spreading of the disease [8, 9] Nevertheless, the available tests are not perfect, errors in sampling and specimen handling can occur, and a patient can have a very low viral load at sampling [10, 11].

There is a report of two cases of medical staff who tested positive for viral RNA during their post-discharge surveillance after clinical recovery from COVID-19 [12]. This report involved only two patients and a detailed analysis was not conducted, but it highlighted a concern for healthcare workers globally. Yin et al. [13] analyzed the clinical characteristics of 58 patients with COVID-19 who were finally diagnosed after multiple negative nucleic acid amplification test (NAAT) results, which showed that multi-site specimens could be taken to reduce the number of missed diagnoses of patients with multiple nucleic acid negatives. Moreover, no studies have focused on understanding the clinical characteristics of patients with recurrent positive NAAT results, and it is unknown whether they are false negatives that finally turn positive, recurrences, or reinfections [14–17]. A better understanding of these cases can facilitate the adjustment of the current discharge criteria, identification of patients with specific pre-existing conditions affecting retest results, and the determination of predictive factors for recurrent positive NAAT results. These points are critical for the development of epidemic control guidelines globally.

Since positive retest results for COVID-19 represent a public health concern because of the increased risk of transmission, this retrospective study comprehensively assessed 184 patients with COVID-19 grouped according to the number of times they tested positive into the negative (positive just once) and positive (positive on more than one test) retest groups. The results of this study can help identify the risk factors for positive retest results.

## Materials and methods

### Study design and patients

This retrospective study examined the data of patients with COVID-19 admitted and treated at the Guanggu District of the Hubei Maternal and Child Health Hospital between February 17 and March 28, 2020. The diagnostic criteria for patient classification were based on the diagnosis and treatment protocol for COVID-19 pneumonia (4th edition) [18]. The study was approved by the ethics committee of the Hubei Maternal and Child Health Hospital (#FYGG(L)2020-004), and written informed consent was obtained from all the patients. The study was performed in adherence to the principles of the Declaration of Helsinki.

### Data collection

The patients’ basic information, clinical manifestations, laboratory test results, and imaging data were obtained from their electronic medical records by the attending physicians. All laboratory test results were measured during the first hospitalization. The drugs for COVID-19 patients were determined by the clinicians. There are no definitive treatments for COVID-19 patients, and the specific medications selected were adopted by clinicians based on a combination of the patient’s condition and the latest guidelines at the time [2–4, 19]. Chronic respiratory diseases include asthma, chronic obstructive pulmonary disease (COPD), bronchiectasis, and other non-acute chronic lung diseases.

### NAAT for SARS-CoV-2

After treatment, the patient was discharged after two consecutive negative NAATs [19, 20]. If a patient had a relapse of the symptoms, NAAT was repeated. At this time, the patients were re-hospitalized because of the positive NAAT; these patients were considered repeatedly positive patients. Nasopharyngeal swabs used for NAAT were collected by the professional medical staff. The samples were analyzed using the 2019-nCoV nucleic acid detection kit (fluorescence-based PCR technique) produced by Shanghai Zhijiang Biotechnology Co., Ltd. (Shanghai, China). This kit uses reverse transcription-polymerase chain reaction (RT-PCR) combined with TaqMan technology and specific primers to detect viral RNA sequences. The test results were interpreted based on the instructions by the manufacturer of the kit. For indeterminate results, the specimens were collected and tested for a second time.

### Statistical analysis

SPSS 23.0 (IBM Corp., Armonk, NY, USA) was used to conduct all statistical analyses in this study. The continuous data were presented as mean ± standard deviation (SD). For inter-group comparisons of continuous variables, the independent *t* test was used for normally distributed datasets and the non-parametric rank-sum test was used for non-normally distributed datasets. Discrete data were described using frequency, and the Chi-square test was used for inter-group comparisons. Univariate logistic regression was used to analyze the significantly different variables, and multivariable binary logistic regression was used to analyze the symptoms and laboratory test results that showed statistical significance during univariate logistic regression. *P* < 0.05 denoted statistical significance for all two-tailed tests.

## Results

### Characteristics of the patients

A total of 184 COVID-19 patients were included in this study; 81 (44.02%) were men. They had a mean age of 59.76 ± 14.38 years, mean height of 162.54 ± 6.43 cm, and mean weight of 62.25 ± 10.60 kg. The positive retest group included 51 patients (27.71%), including 20 men (39.21%) and 31 women (60.78%). The negative retest group was comprised of 133 patients (72.28%), including 61 men (45.86%) and 72 women (54.13%). There were no significant differences in sex, height, weight, age, number of family members, living space, or duration of exercise between the two groups (*P* > 0.05) (Table 1).

**Table 1:** Characteristics of the patients in the positive retest group and negative retest group

Feature	All	Negative retest group	Positive retest group	*P*
Age (years), mean ± SD (*n*)	59.76 ± 14.38 (184)	60.34 ± 14.04 (133)	58.24 ± 15.25 (51)	0.703
Men, *n* (%)	81 (44.02%)	61 (45.86%)	20 (39.22%)	0.416
Height (cm), mean ± SD (*n*)	162.54 ± 6.43 (56)	162.67 ± 5.89 (39)	162.24 ± 7.72 (17)	0.801
Weight (kg), mean ± SD (*n*)	62.25 ± 10.60 (56)	62.41 ± 10.43 (39)	61.88 ± 11.30 (17)	0.915
Number of family members, mean ± SD (*n*)	3.54 ± 1.59 (50)	3.65 ± 1.70 (34)	3.31 ± 1.35 (16)	0.517
Housing area (m^2^), mean ± SD (*n*)	97.98 ± 27.71 (41)	94.19 ± 24.12 (26)	104.53 ± 32.90 (15)	0.362
Exercise				0.682
No exercise, *n* (%)	18 (42.86%)	12 (42.86%)	6 (42.86%)	
≤ 1 h/day, *n* (%)	16 (38.10%)	12 (42.86%)	4 (28.57%)	
1–2 h/day, *n* (%)	4 (9.52%)	2 (7.14%)	2 (14.29%)	
≥ 2 h/day, *n* (%)	4 (9.52%)	2 (7.14%)	2 (14.29%)	
Hypertension, *n* (%)	24 (13.04%)	18 (13.53%)	6 (11.76%)	0.750
Diabetes (type 1 and type 2 diabetes), *n* (%)	8 (4.35%)	5 (3.76%)	3 (5.88%)	0.527
Cardiovascular disease, *n* (%)	9 (4.89%)	7 (5.26%)	2 (3.92%)	0.706
Cerebrovascular disease, *n* (%)	2 (1.09%)	1 (0.75%)	1 (1.96%)	0.479
Chronic respiratory disease, *n* (%)	7 (3.80%)	6 (4.51%)	1 (1.96%)	0.769
Coexistence of 2 or more comorbidities, *n* (%)	28 (15.22%)	19 (14.29%)	9 (17.56%)	0.570
Highest temperature (°C), mean ± SD (*n*)	36.97 ± 0.91 (154)	36.95 ± 0.89 (112)	37.03 ± 0.95 (42)	0.751
Heart rate (bpm), mean ± SD (*n*)	84.85 ± 12.23 (172)	84.58 ± 11.45 (129)	85.65 ± 14.42 (43)	0.880
Pulse oxygen saturation (%), mean ± SD (*n*)	97.10 ± 2.53 (49)	97.30 ± 2.21 (46)	94.00 ± 5.29 (3)	0.122
The overall course (days), mean ± SD (*n*)	48.02 ± 13.45 (161)	50.45 ± 12.71 (110)	42.78 ± 13.61 (51)	0.001
Hospitalization days, mean ± SD (*n*)	21.05 ± 10.31 (174)	24.00 ± 10.18 (123)	13.94 ± 6.49 (51)	< 0.001
Days from onset to hospitalization, mean ± SD (*n*)	15.47 ± 11.05 (157)	17.79 ± 10.37 (108)	10.35 ± 10.86 (49)	< 0.001
Days from onset to the first nucleic acid positive, mean ± SD (*n*)	17.98 ± 16.08 (91)	20.81 ± 15.14 (43)	15.44 ± 16.62 (48)	0.033
Number of negative nucleic acid tests (until the first positive), mean ± SD (*n*)	2.91 ± 1.22 (181)	2.81 ± 1.23 (133)	3.19 ± 1.14 (48)	0.037

The n in brackets represents the number of subjects with data

### Comorbidities

Of the 184 patients, 78 (42.39%) had pre-existing conditions: 24 had hypertension (13.04%), 9 had cardiovascular disease (4.89%), 8 had diabetes (4.35%), 2 had cerebrovascular disease (1.09%), 7 had chronic respiratory disease (3.80%), and 28 had two or more comorbidities (15.22%). In the positive retest group, 22 patients had comorbidities (43.13%): 6 had hypertension, 3 had diabetes, 2 had cardiovascular disease, 1 had cerebrovascular disease, 1 had chronic respiratory disease, and 9 had two or more comorbidities. In the negative retest group, 56 patients had pre-existing conditions (42.10%): 18 had hypertension, 5 had diabetes, 7 had cardiovascular disease, 1 had cerebrovascular disease, 6 had chronic respiratory disease, and 19 had two or more comorbidities. There were no significant differences between the two groups related to comorbidities (Table 1).

### Course of disease, hospital stay, and NAAT

The average course of disease for all the patients was 48.02 ± 13.45 days. The overall duration of disease was 50.45 ± 12.71 days for the negative retest group and 42.78 ± 13.61 days for the positive retest group (*P* = 0.001). The sensitivity of the overall duration of the disease was 100.0%; specificity was 64.70%, and the likelihood ratio was 2.83. The average durations of hospital stay were 21.05 ± 10.31 days for all the patients, 24.00 ± 10.18 days for the negative retest group, and 13.94 ± 6.49 days for the positive retest group (*P* < 0.001). The sensitivity of the number of days of hospitalization was 100.00%; specificity was 47.10%, and the likelihood ratio was 1.89. The average duration from symptom onset to hospitalization was 15.47 ± 11.05 days for all patients, 17.79 ± 10.37 days for the negative retest group, and 10.35 ± 10.86 days for the positive retest group (*P* < 0.001). The sensitivity of the duration from onset to hospitalization was 50.00%; specificity was 82.40% and the likelihood ratio was 2.84. The average duration from symptom onset to the first positive NAAT result was 17.98 ± 16.08 days for all patients, 20.81 ± 15.14 days for the negative retest group, and 15.44 ± 16.62 days for the positive retest group (*P* = 0.033). The sensitivity of the duration from symptom onset to the first positive NAAT result was 50.00%; specificity was 94.10%, and the likelihood ratio was 8.47. On average, the number of negative results before the first positive result was 2.91 ± 1.22% for all the patients, 2.81 ± 1.23 for the negative retest group, and 3.19 ± 1.14 for the positive retest group (*P* = 0.037). The sensitivity of the number of negative nucleic acid tests (until the first positive) was 0.10%; the specificity was 91.7%, and the likelihood ratio was 1.18 (Tables 1, 2).

**Table 2:** Sensitivity, specificity, and likelihood ratio of statistically significant variables (excluding treatment-related variables)

Variables	Sensitivity (%)	Specificity (%)	Likelihood ratio
Hospitalization days	100.00	47.10	1.89
Overall course	100.00	64.70	2.83
Onset to hospitalization	50.00	82.40	2.84
Time from symptom onset to the first positive NAAT result	50.00	94.10	8.47
Number of negative nucleic acid tests (until the first positive)	0.10	91.70	1.18
Cough	52.90	78.20	2.43
Dyspnea	86.30	39.80	1.43
Ground-glass opacities	82.40	40.60	1.39
Bilateral lung infection	5.90	97.00	1.97
Inflammatory absorption	5.90	98.50	3.93
Basophilic	50.00	52.90	1.06
Total protein	100.00	5.90	1.06
Globulin	9.10	96.00	2.28
Direct bilirubin	50.00	64.70	1.42
Potassium	50.00	94.10	8.47
Chlorine	100.00	0.00	1.00
APTT	100.00	23.50	1.31

### Clinical signs and symptoms

Of the 184 COVID-19 patients in this study, 113 had a fever (61.41%), 56 had a cough (30.43%), 98 experienced fatigue (53.26%), 63 had muscle pain (34.24%), 48 had dyspnea (26.09%), and 132 had poor appetite (71.74%). In the positive retest group, 27 patients presented with a cough (52.94%), while 29 in the negative retest group presented with a cough (21.80%) (*P* < 0.001). The sensitivity of cough was 52.90%; specificity was 78.20%, and the likelihood ratio was 2.43. Seven patients in the positive retest group had dyspnea (13.73%), in contrast with 41 in the negative retest group (30.83%) (*P* = 0.018). The sensitivity of dyspnea was 86.30%; specificity was 39.80%, and the likelihood ratio was 1.43 (Tables 2, 3).

**Table 3:** Comparison of the clinical symptoms and computed tomography imaging of lung between two groups

Feature	Total (*n* = 184)	Negative retest group (*n* = 133)	Positive retest group (*n* = 51)	*P*
Fever, *n* (%)	113 (61.41)	84 (63.16)	29 (56.86)	0.432
Cough, *n* (%)	56 (30.43)	29 (21.80)	27 (52.94)	< 0.001
Hemoptysis, *n* (%)	1 (0.54)	1 (0.75)	0 (0.00)	0.535
Chest tightness, *n* (%)	35 (19.02)	23 (17.29)	12 (23.53)	0.335
Chest pain, *n* (%)	8 (4.35)	6 (4.51)	2 (3.92)	0.861
Nasal congestion, *n* (%)	1 (0.54)	1 (0.75)	0 (0.00)	0.535
Runny nose, *n* (%)	1 (0.54)	1 (0.75)	0 (0.00)	0.535
Sore throat, *n* (%)	9 (4.89)	8 (6.02)	1 (1.96)	0.254
Fatigue, *n* (%)	98 (53.26)	69 (51.88)	29 (56.86)	0.544
Muscle aches, *n* (%)	63 (34.24)	48 (36.09)	15 (29.41)	0.393
Drowsiness, *n* (%)	1 (0.54)	1 (0.75)	0 (0.00)	0.535
Headache, *n* (%)	5 (2.72)	3 (2.26)	2 (3.92)	0.534
Dyspnea, *n* (%)	48 (26.09)	41 (30.83)	7 (13.73)	0.018
Abdominal pain, *n* (%)	3 (1.63)	2 (1.50)	1 (1.96)	0.827
Diarrhea, *n* (%)	15 (8.15)	10 (7.52)	5 (9.80)	0.612
Nausea, *n* (%)	3 (1.63)	1 (0.75)	2 (3.92)	0.129
Vomiting, *n* (%)	5 (2.72)	2 (1.50)	3 (5.88)	0.102
Poor appetite, *n* (%)	132 (71.74)	100 (75.19)	32 (62.75)	0.057
Ground-glass opacity, *n* (%)	121 (65.76)	79 (59.40)	42 (82.35)	0.003
Consolidation, *n* (%)	7 (3.80)	4 (3.01)	3 (5.88)	0.362
Mixed manifestation, *n* (%)	5 (2.72)	2 (1.50)	3 (5.88)	0.102
The left lung infection, *n* (%)	7 (3.80)	6 (4.51)	1 (1.96)	0.418
The right lung infection, *n* (%)	10 (5.43)	8 (6.02)	2 (3.92)	0.575
Bilateral lung infection, *n* (%)	111 (60.33)	69 (51.88)	42 (82.35)	< 0.001
Inflammatory absorption, *n* (%)	86 (46.74)	47 (35.34)	39 (76.47)	< 0.001

### Chest CT scan features

Of the 184 patients, 65.76% had ground-glass opacities, 60.33% had bilateral lung infection, 9.23% had unilateral lung infection, and 46.74% showed inflammatory exudate absorption on their chest CT scan. In addition, 59.40% of the negative retest group and 82.35% of the positive retest group showed ground-glass opacities (*P* = 0.003). The sensitivity of ground-glass opacities was 82.40%; the specificity was 40.60%, and the likelihood ratio was 1.39. Bilateral lung infection was found in 51.88% of the patients in the negative retest group and 82.35% of patients in the positive retest group (*P* < 0.001). The sensitivity of bilateral lung infection was 5.90%; specificity was 97.00%, and the likelihood ratio was 1.97. In the negative retest group, 35.34% of the patients showed inflammatory exudate absorption on their CT scans, in contrast with 76.47% of the patients in the positive retest group (*P* < 0.001). The sensitivity of inflammatory exudate absorption was 5.90%; specificity was 98.50%, and the likelihood ratio was 3.93 (Tables 2, 3).

### Laboratory tests

The mean basophil count was 0.03 ± 0.02 × 10^9^ cells/L in the positive retest group and 0.02 ± 0.01 × 10^9^ cells/L in the negative retest group (*P* = 0.023). The sensitivity of the basophil count was 50.00%; specificity was 52.90%, and the likelihood ratio was 1.06. The mean serum total protein concentration was 68.73 ± 6.01 g/L in the negative retest group and 71.79 ± 6.71 g/L in the positive retest group (*P* = 0.014). The sensitivity of serum total protein concentration was 100%; specificity was 5.90%, and the likelihood ratio was 1.06. The mean globulin concentration was 30.17 ± 4.02 g/L in the negative retest group and 32.97 ± 4.46 g/L in the positive retest group (*P* = 0.001). The sensitivity of the globulin concentration was 9.10%; specificity was 96.00%, and the likelihood ratio was 2.28. The mean serum direct bilirubin was 4.05 ± 1.68 μmol/L in the negative retest group and 5.06 ± 2.47 μmol/L in the positive retest group (*P* = 0.032). The sensitivity of the direct bilirubin concentration was 50.00%; specificity was 64.70%, and the likelihood ratio was 1.42. Significant differences between the negative and positive retest groups were observed for the potassium (4.45 ± 0.67 vs. 4.07 ± 0.51 mmol/L, *P* = 0.001) and chlorine (104.76 ± 2.63 vs. 105.93 ± 2.16 mmol/L, *P* = 0.023) concentrations and the activated partial thromboplastin time (APTT) (31.71 ± 5.26 vs. 33.26 ± 3.86, *P* = 0.045). The sensitivities of the serum potassium and chloride concentrations and the APTT were 50%, 100%, and 100%, respectively. Their specificities were 94.10%, 0%, and 23.5%, and their likelihood ratios were 8.47, 1, and 1.31, respectively (Tables 2, 4).

**Table 4:** Comparison of the laboratory examinations between two groups

Characteristic	The negative on retest group	The positive on retest group	*P*
White blood cells (× 10^9^/L), mean ± SD (*n*)	5.99 ± 2.28 (127)	6.04 ± 1.53 (35)	0.427
Red blood cells (× 10^12^/L), mean ± SD (*n*)	4.20 ± 0.56 (127)	4.20 ± 0.65 (36)	0.980
Hemoglobin (g/L), mean ± SD (*n*)	129.60 ± 20.38 (127)	129.11 ± 20.08 (35)	0.364
Platelets (× 10^9^/L), mean ± SD (*n*)	227.36 ± 73.62 (127)	217.41 ± 66.32 (34)	0.455
Neutrophil granulocytes (× 10^9^/L), mean ± SD (*n*)	3.83 ± 2.19 (127)	3.75 ± 1.33 (33)	0.614
Neutrophil granulocytes (%), mean ± SD (*n*)	61.50 ± 11.26 (127)	61.42 ± 9.84 (33)	0.970
Lymphocytes (× 10^9^/L), mean ± SD (*n*)	1.55 ± 0.48 (128)	1.71 ± 0.61 (37)	0.173
Lymphocytes (%), mean ± SD (*n*)	27.88 ± 8.76 (128)	29.39 ± 9.36 (37)	0.376
Monocytes (× 10^9^/L), mean ± SD (*n*)	0.44 ± 0.52 (127)	0.41 ± 0.14 (34)	0.714
Monocytes (%), mean ± SD (*n*)	7.04 ± 2.37 (127)	7.02 ± 2.14 (34)	0.937
Eosnophils (× 10^9^/L), mean ± SD (*n*)	0.15 ± 0.15 (127)	0.13 ± 0.08 (34)	0.659
Eosnophils (%), mean ± SD (*n*)	2.66 ± 2.52 (127)	2.74 ± 3.65 (34)	0.962
Basophils (× 10^9^/L), mean ± SD (*n*)	0.02 ± 0.01 (127)	0.03 ± 0.02 (33)	0.023
Basophils (%), mean ± SD (*n*)	0.45 ± 0.64 (127)	0.42 ± 0.23 (33)	0.082
C-reactive protein (mg/dL), mean ± SD (*n*)	11.50 ± 29.59 (116)	5.69 ± 15.25 (31)	0.259
Fasting blood glucose (mmol/L), mean ± SD (*n*)	5.59 ± 1.92 (95)	5.85 ± 2.83 (24)	0.631
Serum total protein (g/L), mean ± SD (*n*)	68.73 ± 6.01 (108)	71.79 ± 6.71 (33)	0.014
Albumin (g/L), mean ± SD (*n*)	38.96 ± 5.36 (110)	39.08 ± 4.02 (33)	0.998
Globulin (g/L), mean ± SD (*n*)	30.17 ± 4.02 (95)	32.97 ± 4.46 (32)	0.001
Total bilirubin (µmol/L), mean ± SD (*n*)	10.23 ± 5.21 (110)	11.58 ± 5.73 (32)	0.260
Direct bilirubin (µmol/L), mean ± SD (*n*)	4.05 ± 1.68 (110)	5.06 ± 2.47 (32)	0.032
ALT (U/L), mean ± SD (*n*)	29.65 ± 25.68 (110)	27.99 ± 30.39 (33)	0.395
AST (U/L), mean ± SD (*n*)	21.32 ± 12.86 (110)	22.92 ± 27.44 (33)	0.463
ALP (U/L), mean ± SD (*n*)	78.39 ± 27.25 (111)	76.53 ± 18.75 (32)	0.768
GGT (U/L), mean ± SD (*n*)	35.70 ± 32.68 (111)	31.91 ± 20.64 (32)	0.666
TBA (μmol/L), mean ± SD (*n*)	4.82 ± 4.30 (109)	3.29 ± 2.08 (31)	0.053
Urea nitrogen (mmol/L), mean ± SD (*n*)	4.91 ± 3.13 (95)	4.42 ± 1.11 (30)	0.757
Creatinine (µmol/L), mean ± SD (*n*)	71.47 ± 53.23 (97)	65.01 ± 10.74 (29)	0.657
Uric acid (µmol/L), mean ± SD (*n*)	301.80 ± 89.85 (102)	326.70 ± 90.21 (30)	0.108
Potassium, mean ± SD (*n*)	4.45 ± 0.67 (103)	4.07 ± 0.51 (30)	0.001
Sodium, mean ± SD (*n*)	142.68 ± 29.51 (103)	140.89 ± 29.10 (28)	0.116
Chlorine, mean ± SD (*n*)	104.76 ± 2.63 (102)	105.93 ± 2.16 (28)	0.023
Calcium, mean ± SD (*n*)	2.16 ± 0.19 (102)	2.13 ± 0.22 (29)	0.947
Phosphorus, mean ± SD (*n*)	1.13 ± 0.24 (92)	1.18 ± 0.19 (28)	0.366
Prothrombin time (s), mean ± SD (*n*)	11.99 ± 1.87 (98)	11.45 ± 0.88 (24)	0.148
International normalized ratio, mean ± SD (*n*)	1.07 ± 0.16 (98)	1.05 ± 0.08 (22)	0.601
Activated partial thromboplastin time (s), mean ± SD (*n*)	31.71 ± 5.26 (98)	33.26 ± 3.86 (23)	0.045
Fibrinogen (g/L), mean ± SD (*n*)	3.60 ± 1.08 (98)	3.45 ± 0.85 (24)	0.864
Thrombin time (s), mean ± SD (*n*)	15.82 ± 3.19 (98)	15.10 ± 1.61 (22)	0.092
d-Dimer (ng/mL), mean ± SD (*n*)	0.94 ± 1.55 (74)	0.46 ± 0.55 (22)	0.255

Data were displayed as mean ± SD

*ALT* alanine aminotransferase, *AST* aspartate aminotransferase, *ALP* alkaline phosphatase, *GGT* γ-glutamyl transpeptidase, *TBA* total bile acid

### Treatment

Of the 184 patients, 71.20% were treated with Traditional Chinese Medicine (TCM). The rates of TCM decoction treatment were 55.64% for the negative retest group and 21.57% for the positive retest group (*P* < 0.001). The sensitivity of the receipt of TCM decoction treatment was 78.40%; specificity was 55.60%, and the likelihood ratio was 1.77. The rate of oseltamivir phosphate usage was 25.56% for the negative retest group and 1.96% for the test–retest group (*P* < 0.001). The sensitivity of oseltamivir phosphate use was 98.00%; specificity was 25.60%, and the likelihood ratio was 1.32. The average duration of use of antiviral medication by the negative retest group was 8.60 ± 4.18 days, compared with 4.29 ± 4.25 days for the positive retest group (*P* < 0.001). The mean duration of antibiotic treatment was 10.22 ± 4.19 days for the negative retest group and 1.87 ± 3.90 days for the positive retest group (*P* < 0.001). The sensitivity of the average duration of antiviral treatment and the mean course of antibiotic treatment was 100.00%; specificity was 88.20%, and the likelihood ratio was 8.47. The average course of TCM in the negative on retest group was 9.79 ± 4.83 days, compared with 7.00 ± 6.49 days in the positive on retest group (*P* = 0.047). The sensitivity of the TCM course was 81.80%, specificity was 56.00%, and the likelihood ratio was 1.86. The rate of oxygen inhalation therapy was 77.60% in the negative retest group and 46.90% in the test–retest group (*P* = 0.005). The sensitivity of oxygen inhalation therapy was 53.10%, the specificity was 77.60%, and the likelihood ratio was 2.37 (Tables 5, 6).

**Table 5:** Comparison of treatment between the two groups

Treatment	Total (*n* = 184)	Negative on retest group (*n* = 133)	Positive on retest group (*n* = 51)	*P*
Traditional Chinese medicine, *n* (%)	131 (71.20)	97 (72.93)	34 (66.67)	0.401
Lianhua qingwen capsules	95 (51.63)	72 (54.14)	23 (45.10)	0.272
Herbal decoctions	85 (46.20)	74 (55.64)	11 (21.57)	< 0.001
Anti-viral therapy, *n* (%)	113 (61.41)	86 (64.66)	27 (52.94)	0.144
Abidor	62 (33.70)	44 (33.08)	18 (35.29)	0.776
Oseltamivir phosphate capsules	35 (19.02)	34 (25.56)	1 (1.96)	< 0.001
Antibiotics, *n* (%)	51 (27.72)	36 (27.07)	15 (29.41)	0.751
Moxifloxacin	49 (26.63)	36 (27.07)	13 (25.49)	0.828
Azithromycin	1 (0.54)	0 (0.00)	1 (1.96)	0.105
Meropenem	1 (0.54)	1 (0.75)	0 (0.00)	0.535
Days of drugs (d), mean ± SD				
Days of taking Chinese medicine	8.63 ± 5.71	9.79 ± 4.83	7.00 ± 6.49	0.047
Days of antiviral drugs	6.80 ± 4.70	8.60 ± 4.18	4.29 ± 4.25	< 0.001
Days of antibiotics	6.41 ± 5.81	10.22 ± 4.19	1.87 ± 3.90	< 0.001
Oxygen inhalation, *n* (%)	53.0 (65.40)	38.0 (77.60)	15.0 (46.90)	0.005

**Table 6:** Sensitivity, specificity and likelihood ratio of variables where the difference is statistically significant (treatment-related variables)

Variables	Sensitivity (%)	Specificity (%)	Likelihood ratio
Course of antiviral treatment	100.00	88.20	8.47
Course of antibiotic treatment	100.00	88.20	8.47
TCM decoction treatment	78.40	55.60	1.77
Oseltamivir phosphate use	98.00	25.60	1.32
Course of TCM	81.80	56.00	1.86
Oxygen inhalation therapy	53.10	77.60	2.37

### Regression analysis

The significantly different characteristics of the two groups were analyzed using univariable logistic regression. The significant characteristics with *P* < 0.05 in the univariate analyses were included in the multivariable logistic regression. Univariable regression showed that cough, dyspnea and the total protein, globulin, direct bilirubin, potassium, and chloride concentrations were all statistically correlated with a positive retest result (*P* < 0.001, *P* = 0.022, *P* = 0.017, *P* = 0.002, *P* = 0.012, *P* = 0.003, and *P* = 0.025, respectively). Multivariable regression analysis showed that coughing (OR = 7.59, 95% CI 2.28–25.32, *P* = 0.001) and serum chloride concentrations (OR = 1.38, 95% CI 1.08–1.77, *P* = 0.010) were independently associated with recurrent positive NAAT results (Table 7).

**Table 7:** Univariate and multivariable logistic regression analysis of independent risk factors for recurrent positive NAAT results

Variable	Univariate analysis			Multivariable analysis		
	OR	95% CI	*P*	OR	95% CI	*P*
Cough	0.248	0.125–0.493	< 0.001	7.594	2.278–25.322	0.001
Dyspnea	0.357	0.148–0.859	0.022	0.541	0.116–2.515	0.433
Basophilic (× 10^9^/L)	1.270	0.991–1.626	0.059			
Total protein (g/L)	1.086	1.015–1.162	0.017	1.056	0.936–1.191	0.377
Globulin (g/L)	1.168	1.057–1.290	0.002	1.054	0.897–1.237	0.524
Direct bilirubin (µmol/L)	1.284	1.056–1.560	0.012	1.295	0.960–1.747	0.091
Potassium (mmol/L)	0.278	0.120–0.644	0.003	0.487	0.162–1.462	0.199
Chlorine (mmol/L)	1.766	1.074–2.901	0.025	1.382	1.082–1.766	0.010
Activated partial thromboplastin time (s)	1.052	0.970–1.141	0.219			

Univariate logistic regression was performed on significantly different variables, and multivariable binary logistic regression was performed on symptoms and laboratory tests that showed statistical significance in the univariate logistic regression (*P* < 0.05)

*OR* odds ratio, *95% CI* 95% confidence interval

## Discussion

Reports of COVID-19 patients retesting positive on NAAT have drawn significant attention [14–17, 20]. This study explored whether factors other than NAAT contribute to positive retest results. The characteristics of 184 COVID-19 patients were retrospectively analyzed. There was no significant difference in gender between the two groups, which was consistent with the findings of Wan et al. [21]. The mean age of the patients was 59.76 years, and 58.20% were > 60 years old. This is consistent with the study by Yang et al. [22], who postulated that older people aged ≥ 60 years are more susceptible to infection by SARS-CoV-2 and its associated mortality. Moreover, more than half of the patients had pre-existing conditions, including hypertension, diabetes, cardiovascular disease, and chronic respiratory disease. The incidence of COVID-19 is higher among patients with multiple comorbidities. This finding is consistent with the results reported by Zhou et al. [23]. They reported that 48% of the 191 patients enrolled in their study had pre-existing conditions, with the most common being hypertension, diabetes, and coronary heart diseases [23].

The Chinese Diagnosis and Treatment Protocol for COVID-19 Pneumonia (8th edition) states that the incubation period for COVID-19 is generally 3–7 days and can be up to 14 days. This study showed that the average duration of disease in the 184 patients was 48.02 days; the average hospital stay was 21.05 days, and the average duration from symptom onset to the first positive NAAT result was 17.98 days. These results confirmed that COVID-19 had a slow onset, a prolonged duration, and a long incubation period. The predictive model from Lauer et al. [24] estimates that 101 of every 10,000 cases will develop symptoms of COVID-19 after 14 days of isolation. This conclusion is consistent with the results of this study, which demonstrated that the average duration from symptom onset to the first positive NAAT result was 17.98 ± 16.08 days for all patients, 20.81 ± 15.14 days for the negative retest group, and 15.44 ± 16.62 days for the positive retest group. The duration from symptom onset to hospitalization and the duration from the onset of symptoms to the first positive NAAT result were shorter for the positive retest group than for the negative retest group, indicating that patients in the positive retest group had a shorter incubation period and a rapid disease progression.

The average duration of hospital stay of the patients in the positive retest group was only 13.94 days, which was significantly shorter than that of the patients in the negative retest group (24.00 days). This is consistent with the significantly lower patronage of TCM, oseltamivir, antiviral medications, herbal decoctions, and other drugs in the positive retest group than in the negative retest group. We speculated that the patients in the positive retest group were not adequately treated and could still transmit the virus, leading to a positive NAAT retest. Therefore, we recommend that patients with a hospital stay duration of < 2 weeks should stay in isolation after discharge and be monitored to reduce transmission.

The current discharge criteria for COVID-19 patients in China are two consecutive negative NAAT results from respiratory specimens with at least 1 day between the two samplings. However, in this study, 184 patients underwent 2.91 NAATs before the first positive result; the number of NAATs in the negative retest group was 2.81, and the average number of NAATs in the positive retest group was 3.19. To better control the spread of the disease, hospitals should ensure that the discharge criteria are strictly followed, and we recommend obtaining at least three negative NAAT results before discharging a patient combined with the significant relief of clinical symptoms, the significant improvement of ground-glass changes on chest CT, and the significant resolution of inflammation.

The common symptoms of COVID-19 include fever, shortness of breath, fatigue, and dyspnea [2, 25]. The results of this study showed that 61.43% of the participants had fever, 30.43% experienced coughing, 53.26% experienced fatigue, 34.24% experienced muscle pain, 26.09% experienced dyspnea, and 71.74% had poor appetite. The proportion of patients who presented with cough was significantly higher in the positive retest group than in the negative retest group, which may be attributed to the higher SARS-CoV-2 viral load in the positive retest group. Imaging features are of great significance for the diagnosis of COVID-19 [26–28]. The positive retest group showed a higher incidence of bilateral lung infection and ground-glass opacities than the negative retest group. Therefore, COVID-19 patients with bilateral lung infections, ground-glass lesions, and other imaging manifestations should be aware of the possibility of recurrence, and nucleic acid monitoring should be strengthened for them.

Increases in the basophil count are mainly observed in patients with allergies and blood and some infectious diseases, including influenza, smallpox, and tuberculosis [29–31]. Shen et al. [32] found significantly increased basophil counts in patients with H1N1 influenza. In the present study, the basophil count was higher in the positive retest group. The basophil count is easily affected by factors such as the detection instrument, temperature of the environment, and the ratio of anticoagulants and can show a false increase. After eliminating the various confounding factors and the allergic condition, if a patient shows high basophil counts on reexamination under the microscope, there is a high possibility that this patient will have a positive result on retest for viral RNA after being discharged, and the basophil count should be followed up closely during the post-discharge isolation. The concentrations of serum globulin and direct bilirubin were both higher for the positive retest group, implying that patients in the positive retest group had more severe liver damage. The actual condition of the patients needs to be evaluated to determine whether hepatoprotective drugs should be prescribed during treatment for COVID-19.

The electrolytes in human plasma play a decisive role in maintaining the osmotic pressure of extracellular fluids, acid–base balance, and the distribution and transfer of body fluids. Changes in the concentrations of electrolytes cause electrolyte disorders [33, 34]. Abnormal concentrations of potassium and chlorine can cause organ damage and even death [35–37]. SARS-CoV-2 binds to angiotensin-converting enzyme 2 (ACE2), which is its receptor, reduces ACE2 expression, and increases angiotensin II, which can result in increased renal reabsorption of chlorine and potassium excretion and, ultimately, high chloride and low potassium concentrations [38–41]. Our research shows that the chlorine concentrations of patients in the positive retest group were increased, and the regression analysis also indicated that the chloride concentrations help predict the risk of positive retest results. The serum potassium concentrations were significantly lower in the positive retest group. This result further indicated that the virus load in the positive group was higher than that in the negative group. Therefore, COVID-19 patients with electrolyte disturbances should be closely followed up. We also recommend stricter discharge criteria and increased NAAT testing for these patients.

This study offers some valuable insights into COVID-19, but it has some limitations. First, the specimens used for NAAT were obtained using nasopharyngeal swabs, and the detection rates for viral RNA from these types of specimens are still under evaluation. Hase et al. [42] reported a case where the induced sputum specimen tested positive on NAAT while the throat swab collected on the same day tested negative. Factors such as the type of specimen and the part of the body the specimen is collected from may be important causes of positive retests. Second, this was a retrospective study with some missing data, which may have led to inevitable biases. For example, there were extreme data on the number of days of hospitalization, overall course, and the concentrations of total protein and chlorine, with a sensitivity of 100.0%. Chlorine had a specificity of 0.0%, which implies that some data can only be used as limited references.

## Conclusion

There were several differences in the clinical characteristics, laboratory test results, imaging features, and treatment outcomes of the patients in the negative and positive retest groups. These differences are highly predictive of retest outcomes and may be useful for guiding risk assessment and clinical management, especially cough and blood chloride concentrations, according to the multivariable analysis. In addition, our results suggested that patients with a hospital stay duration of < 2 weeks or a short incubation period should stay in isolation and be monitored after discharge to reduce transmission.
